# Prediction and Prognostication of Peripheral Blood Biomarkers in Head and Neck Cutaneous Squamous Cell Carcinoma

**DOI:** 10.1002/ohn.70174

**Published:** 2026-02-24

**Authors:** Mikayla G. Hubbard, Emily H. Chestnut, Meghana C. Bhaskara, Lena W. Chen, Elena Kennedy, Aimee Lee, Jessica Yesensky, Janice L. Farlow

**Affiliations:** ^1^ Indiana University School of Medicine Indianapolis Indiana USA; ^2^ Department of Otolaryngology–Head and Neck Surgery Indianapolis Indiana USA

**Keywords:** cutaneous squamous cell carcinoma of head and neck, peripheral blood biomarkers, prognostic factors, staging systems

## Abstract

**Objective:**

While many with head and neck cutaneous squamous cell carcinomas (cHNSCC) respond well to surgery alone, there is a need to refine traditional staging systems. We aim to identify whether peripheral blood biomarkers (PBBMs) may enhance current staging for prognostication and adjuvant treatment selection.

**Study Design:**

Retrospective cohort study.

**Setting:**

Single, tertiary academic referral center.

**Methods:**

Preoperative and postoperative PBBMs and patient/disease characteristics were collected for adults surgically treated for cHNSCC at an academic center from 2016 to 2022. Pearson correlations and *t*‐tests were utilized to correlate PBBMs with tumor characteristics. Univariate and multivariate analysis was performed to correlate predictors with overall survival. Staging systems were compared using Kaplan‐Meier regression and Chi‐square test for trend and independence.

**Results:**

409 patients (median age 75.4, 77% male, 99% White, median follow‐up 34.5 months) underwent surgical resection for cHNSCC between 2016 and 2022. While several preoperative PBBMs correlated with tumor size and nodal burden, none were found to predict OS. On univariate analysis, high postoperative relative neutrophils and platelet‐to‐lymphocyte ratio (PLR) were associated with worse OS. High relative lymphocytes, total albumin, and prognostic nutritional index (PNI) were associated with improved OS. On multivariate analysis, no PBBM was significant for predicting OS.

**Conclusion:**

This is the first study to evaluate the prognostic value of PBBMs in cHNSCC. Preoperative PBBMs correlated with tumor size and nodal burden, while high CCI and low postoperative NLRs negatively predicted survival. Further prospective research is needed for validation.

Nonmelanoma skin cancer is the most common malignancy globally, with cutaneous squamous cell carcinoma (cSCC) being the second‐most common malignancy in White individuals.^1^ Repetitive sun exposure is a known risk factor for the development of skin malignancies, and as such, the head and neck are the most common sites for cSCC to develop.^2^ Most patients with cSCC present with early‐stage disease where minimal surgical resection or cryotherapy is sufficient for disease eradication; however, traditional tumor staging systems for cSCC of the head and neck (cHNSCC) are suboptimal and fail to consistently identify when more aggressive surgical intervention or adjuvant therapy is warranted.^3^, ^4^, ^5^, ^6^

Previous systematic reviews have revealed that tumor depth, margins of primary tumor, number of lymph nodes affected, parotid disease involvement, age, and states of immunosuppression are significant prognostic factors in staging and treating cSCC; however, other biomarkers beyond patient and histopathological data have not been clearly established for cHNSCC.^2^, ^7^ Peripheral blood biomarkers (PBBMs) are a pertinent area of study in cancer research given they are easily accessible, cost‐efficient, effective for longitudinal disease management, and personalized to the patient. While PBBMs have been previously studied in head and neck cancer, these studies either focus solely on mucosal HNSCC, or they do not stratify cHNSCC from the other head and neck cancers that may affect nutritional intake given mucosal primary sites, bringing into question their generalizability to cHNSCC. Several studies have demonstrated perioperative PBBMs are significant for predicting survival in HNSCC.^8^, ^9^, ^10^ Pan et al also found that lower baseline relative lymphocytes and higher levels of serum lactate dehydrogenase correlated with increased resistance to immune checkpoint inhibitors.^11^ This study included cHNSCC, but once again did not stratify for it. The current literature on primarily mucosal HNSCC highlights the paucity of identifiable serum markers with prognostic value for cHNSCC and emphasizes the importance of reliable biomarkers to assist risk stratification and treatment strategies.

When staging cSCC, there are three main systems frequently referenced (Table 1). Both the American Joint Committee on Cancer (AJCC) and International Union Against Cancer (UICC) list specific characteristics at each stage, which include tumor size, depth of invasion, perineural and/or bone invasion, and skull base involvement.^12^, ^13^ The Brigham Women's Hospital (BWH) was developed due to inadequate prognostication in current staging systems and utilizes number of risk factors (Table 2) associated with the primary tumor to identify stage.^14^ There is limited information or consensus regarding which staging system most accurately predicts patient outcomes and need for adjuvant therapy.^5^, ^15^, ^16^ The AJCC is the most commonly used and clinician‐recognized staging system to attempt prognostication based on tumor characteristics; however, previous studies have found it may not be the superior system, despite its recognition.^14^ Karia et al revealed BWH staging system performed superiorly in their cohort and demonstrated 4 (T1‐T3) statistically distinct categories with identifiable risk increases per stepwise category advancement.^15^, ^17^

**Table 1:** T Category Criteria for Cutaneous Squamous Cell Carcinoma by the Three Major Staging Systems: 8th Edition of the American Joint Committee on Cancer (AJCC), Union for International Cancer Control (UICC), and Brigham and Women's Hospital (BWH)

8th Ed. AJCC staging criteria		UICC staging criteria		BWH staging criteria^a^	
Cat.	Criteria	Cat.	Criteria	Cat.	Criteria
T1	Tumor <2 cm in greatest dimension	T1	Tumor ≤2 cm in greatest dimension	T1	0 risk factors
T2	Tumor ≥2 cm, but <4 cm in greatest dimension	T2	Tumor >2 cm in greatest dimension	T2a	1 risk factor
T3	Tumor has one or more of the following characteristics:	T3	Tumor with invasion of deep structures (muscle, cartilage, upper or lower extremity bone, orbit)	T2b	2‐3 risk factors
	≥4 cm in greatest dimension				
	Minor bone erosion				
	Perineural Invasion^b^				
	Deep Invasion^c^				
T4a	Tumor with gross cortical bone/marrow invasion	T4	Tumor with invasion of axial skeleton or direct perineural invasion of the skull base	T3	4 risk factors or bone invasion
T4b	Tumor with skull base invasion and/or skull base foramen involvement.				

^a^ Specific high‐risk features determined by staging system can be found in Table 2.

^b^ Perineural invasion is defined as tumor cells present in the nerve sheath of a nerve in one of three characteristics: (1) lying deeper than the dermis, (2) measuring ≥1 mm in caliber, or (3) presenting with clinical/radiographic involvement of named nerves without skull base invasion or transgression.

^c^ Deep invasion is defined as invasion beyond the subcutaneous fat or >6 mm (measured from the granular layer of the adjacent normal epidermis to the base of tumor.

**Table 2:** High‐Risk Features of Cutaneous Squamous Cell Carcinoma, defined by Staging System

Category	Classically defined high‐risk features	7th Ed. AJCC high‐risk features*	BWH high‐risk features
Size/location and patient demographics	Tumor ≥2 cm at greatest diameter	Tumor size >2 cm	Clinical tumor diameter ≥2 cm
	Location on ear or lip	Location on the ear or lip	
	Recurrence	Depth >2 mm or Clark level ≥IV	
	Patient immunosuppression		
Depth	Tumor extends beyond dermis	Depth >2 mm or Clark level ≥IV	Tumor invasion beyond the subcutaneous fat
	Perineural Invasion	Perineural invasion	Perineural invasion of nerve(s) ≥0.1 mm in caliber
Grading	Poorly differentiated histology	Poorly differentiated histology	Poorly differentiated histology

* The AJCC 7th Edition is no longer used as it is outdated by the 8th Edition; however, risk factors identified could yield relevance.

In this study, we attempt to parallel the work done for mucosal HNSCC by identifying predictive and prognostic PBBMs pre‐ and postoperatively that could be utilized clinically to more clearly determine survival and adjuvant therapy needs in cHNSCC cases. We also sought to compare this to the relative prognostic performance of the most commonly used staging systems.

## Methods

### Study Design and Demographic Information Collection

A retrospective cohort study was performed at a single academic institution (Indiana University Institutional Review Board 23448) on adult patients (≥18 years of age) with surgically treated cHNSCC between January 2016 and December 2022. Patients with unknown primary tumor data, presumed metastatic SCC disease with unknown primary source or concurrent solid tumor cancer at the time of operation were excluded from the study. Demographics including age at time of operation, sex, race and ethnicity, zip code, tobacco and alcohol use history, and presence of specific comorbidities using the Charlson Comorbidity Index were collected.^18^ History of chronic immunosuppression was also collected, which was defined as long‐term use of immunosuppressant agents for autoimmune conditions or solid organ transplants, as well as immunosuppressed diseases, like chronic leukemia, human immunodeficiency virus, or other leukocyte dysregulating diseases.

The primary endpoint for this study was correlating overall survival (OS) to preoperative and postoperative PBBM data. Secondary endpoints of this study included determining significant preoperative PBBMs that can be utilized to predict high‐risk intraoperative findings and comparing the T category per the three main staging systems with our cohort's survival outcomes.

### Peripheral Blood Biomarkers (PBBMs) and Collected Data for Analysis

Of the 409 patients included in this study, 274 (67.0%) had preoperative labs (median 27 days) and 257 (62.8%) had postoperative labs (median 46 days) collected within the included time frame (±6 months from operation). If a patient had multiple lab values within this timeframe, the lab values closest to their date of operation were selected. PBBMs collected for the purposes of this study include relative and absolute neutrophils, lymphocytes, monocytes, and eosinophils; platelets; and total albumin. Platelet‐to‐lymphocyte ratio (PLR), neutrophil‐to‐lymphocyte ratio (NLR), and Prognostic Nutritional Index (PNI) were calculated from the collected PBBMs. PNI was defined per the literature as follows: PNI = 10*(serum albumin, g/dL) + 0.005*(total lymphocyte count per mm^3^).^19^

Intraoperative findings collected included location of primary tumor, greatest dimension (cm), depth of invasion (in situ, to dermis, to subcutis, to deep structures), histologic subtype, perineural involvement, bone erosion (macro or micro involvement), absolute metastatic lymph nodes resected, and metastatic‐to‐total lymph node ratio. Deep invasion was defined by the AJCC as invasion beyond the subcutaneous fat or >6 mm (measured from the granular layer of the adjacent normal epidermis to the base of tumor).^12^ Perineural invasion is described by the AJCC as meeting one of three criteria: found in a layer deeper than the dermis, measuring ≥1 mm in caliber, or presenting with clinical/radiographic involvement of named nerves without skull base invasion or transgression.^12^

### Statistical Analysis

Descriptive statistics were calculated for demographics and oncologic characteristics. Pearson correlation was applied to continuous intraoperative findings (tumor size and metastatic burden at time or surgery. To analyze preoperative PBBMs to grouped variables (presence or absence of deep invasion and perineural invasion), an unpaired, two‐tailed *t*‐test was utilized with the False‐Discovery Rate (FDR) set to 1% using the two‐stage step‐up method. To estimate OS of our cohort based on the preoperative and postoperative PBBMs collected, we utilized both univariate and multivariate Cox Regression. Univariate analyses were conducted between age, CCI, and preoperative and postoperative PBBMs regarding OS without adjustment; multivariate regressions were chosen based on discovery on the univariate level at *P* < .1 and adjusted for history of tobacco use, history of chronic immunosuppression, and stage. To explore potential confounding between subjects who did and did not receive preoperative and postoperative laboratory testing, we compared OS between the groups using a Kaplan‐Meier curve with Log‐rank Mantel‐Cox Regression. To compare our cohort's survival against the three staging systems, a Kaplan‐Meier curve was constructed with Log‐rank Mantel‐Cox Regression to determine significance between each system's stages. To determine homogeneity (demonstrating similar outcomes for patients with same‐stage disease) and monotonicity (demonstrating a stepwise progression of worse outcomes with more severe disease) of the staging systems, a Chi‐square test for independence and for trend were utilized, respectively. A *P* < .05 was considered statistically significant and 95% confidence intervals were reported if indicated. All statistics were performed using GraphPad Prism 10 2025.

## Results

### Patient and Disease Characteristics

This study included 409 patients (median age 75, range 40‐101 years) who underwent surgical resection of cHNSCC between 2016 and 2022. Of these, 317 (77.1%) were male, and 406 (98.8%) were non‐Hispanic/Latino White (Table 3). Median follow‐up duration was 34.5 months (range 0‐114.4 months). Sixty‐seven (16.4%) had a history of chronic immunosuppression. The median Charlson Comorbidity Index (CCI) was 7.0 (range 1.0‐19.0). The most common site for primary tumor was various locations of the face (176 patients, 43.0%); second most common was the ear and temporal region (156 patients, 38.1%).

**Table 3:** Patient and Disease Characteristics

Patient and disease characteristics	*N* = *409 (%)*
Age	
Median, years ± standard deviation (range)	75.4 ± 11.4 (40‐101)
Hazard Ratio (95% CI, *P*‐value)	1.039 (1.024‐1.054, <.0001)
Sex	
Male	315 (77.0%)
Female	94 (23.0%)
Race	
Non‐Hispanic/Latino White	406 (99.2%)
Hispanic and/or Latino	2 (0.5%)
Unknown	1 (0.2%)
Charlson Comorbidity Index (CCI) Score Median (range)	7.0 (1.0‐19.0)
≤5	104 (25.4%)
6‐10	236 (57.7%)
>10	69 (16.9%)
Hazard ratio (95% CI, *P*‐value)	1.217 (1.164‐1.270, <.0001)
History of chronic immunosuppression	
Yes	67 (16.4%)
No	342 (83.6%)
Primary tumor site	
Face	176 (43.0%)
Cheek	89
Forehead or scalp	73
Chin	2
Unspecified region of face	11
Ear or temporal region	156 (38.1%)
Neck	28 (6.8%)
Nose	23 (5.6%)
Lip	13 (3.2%)
Other/unspecified region of head or neck	12 (2.9%)
Deep invasion	
Yes	125 (30.6%)
No	284 (69.4%)
Metastatic disease at time of primary surgery	
Positive lymph nodes during resection	137 (33.5%)
Perioperative therapy	
Preoperative radiation and/or chemotherapy	21 (5.1%)
Postoperative radiation and/or chemotherapy	101 (24.7%)

Abbreviation: CI, confidence interval.

### Analyses of Preoperative PBBMs and Intraoperative Disease Findings

Tumor size was significantly correlated to relative and absolute neutrophil (*r* = 0.148 and 0.324, respectively), absolute lymphocytes (*r* = 0.220), absolute monocytes (*r* = 0.152), and PLR (Table 4A). Furthermore, it was negatively correlated to total albumin (*r* = −0.158). Total number of metastatic lymph nodes was only correlated to relative monocytes (*r* = 0.391), while metastatic‐to‐total lymph node ratio was correlated to PLR (*r* = 0.367) (Table 4B,C).

**Table 4:** Predicting Tumor Size and Metastatic Lymph Nodes with Preoperative Peripheral Blood Biomarkers

	(A) Tumor size			(B) Total metastatic lymph nodes			(C) Metastatic‐to‐total lymph nodes		
PBBM	r	95% CI	*P*‐value	r	95% CI	*P*‐value	r	95% CI	*P*‐value
Relative neutrophils	0.148	0.000 to 0.289	.049	−0.031	−0.277 to 0.209	.753	0.120	−0.124 to 0.350	.334
Absolute neutrophils	0.324	0.186 to 0.450	<.0001	−0.005	−0.245 to 0.235	.965	0.029	−0.213 to 0.267	.816
Relative lymphocytes	−0.003	−0.151 to 0.144	.964	0.004	−0.236 to 0.244	.973	−0.122	−0.352 to 0.121	.324
Absolute lymphocytes	0.220	0.075 to 0.355	.003	−0.049	−0.286 to 0.194	.696	−0.080	−0.314 to 0.163	.519
Relative monocytes	−0.009	−0.156 to 0.139	.909	0.391	0.167 to 0.577	.011	0.204	−0.038 to 0.423	.098
Absolute monocytes	0.152	0.005 to 0.293	.043	−0.036	−0.274 to 0.206	.771	−0.045	−0.282 to 0.198	.721
Relative eosinophils	−0.069	−0.216 to 0.080	.361	0.231	−0.014 to 0.450	.064	0.045	−0.201 to 0.286	.721
Absolute eosinophils	0.101	−0.048 to 0.246	.182	0.199	−0.047 to 0.422	.112	0.001	−0.243 to 0.245	.992
Platelets	0.224	0.100 to 0.341	.0005	0.089	−0.103 to 0.277	.361	0.099	−0.094 to 0.286	.313
Total albumin	−0.158	−0.289 to −0.020	.025	−0.025	−0.239 to 0.192	.821	0.040	−0.178 to 0.253	.721
PNI	−0.072	−0.217 to 0.076	.339	0.010	−0.228 to 0.246	.937	−0.014	−0.250 to 0.224	.911
PLR	0.197	0.051 to 0.335	.009	−0.048	−0.285 to 0.195	.701	0.367	0.139 to 0.558	.002
NLR	0.157	0.009 to 0.298	.037	−0.023	−0.264 to 0.220	.853	0.236	−0.006 to 0.452	.056

Abbreviations: CI, confidence interval; NLR, neutrophil‐to‐lymphocyte ratio; PLR, platelet‐to‐lymphocyte ratio; PNI, Prognostic Nutritional Index.

For tumors separately exhibiting deep invasion and perineural invasion, preoperative absolute neutrophils (*P* = .002, *P* = .011, respectively) and platelets (*P* = .014, *P* = .035, respectively) were increased although they did not reach significance after FDR corrections (Table 5).

**Table 5:** Predicting Deep Invasion and PNI with Preoperative Peripheral Blood Biomarkers

	(A) Deep invasion			(B) Perineural invasion		
PBBM	*P*‐value	Difference of mean^a^	Corrected discovery?^b^	*P*‐value	Difference of mean^a^	Corrected discovery?^b^
Relative neutrophil	.205	−2.43	No	.272	−3.114	No
Absolute neutrophil	**.002**	−1,117	No	**.011**	−1,295	No
Relative lymphocyte	.872	23.06	No	.916	0.354	No
Absolute lymphocyte	.089	2905	No	.640	−450.8	No
Relative monocyte	.191	−12.38	No	.283	5.621	No
Absolute monocyte	.421	−35.96	No	.161	−90.26	No
Relative eosinophil	.407	2.313	No	.425	0.253	No
Absolute eosinophil	.122	−37.13	No	.952	32.24	No
Platelets	**.014**	−28,735	No	**.035**	−34,581	No
Total albumin	.387	−0.03	No	.314	−0.113	No
PNI	.189	−2.73	No	.351	−5.469	No
PLR	.158	−26.64	No	.174	−52.97	No
NLR	.387	−0.54	No	.181	1.432	No

Abbreviations: CI, confidence interval; NLR, neutrophil‐to‐lymphocyte ratio; PLR, platelet‐to‐lymphocyte ratio; PNI, Prognostic Nutritional Index.

^a^ Difference of mean was calculated by subtracting the mean of positive group from the negative group. Negative numbers indicate the positive group had a higher mean. Positive numbers indicate the negative group had a higher mean.

^b^ FDR = 1% for correction.

### Univariate Analysis of PBBMs associated with Survival in cHNSCC

Univariate Cox Regressions were utilized on all collected preoperative and postoperative PBBMs (Table 6). Both age (HR 1.039, *P* < .0001) and CCI (HR 1.217, *P* < .0001) were significant for worse OS on the single variable level. No preoperative PBBMs were significant for predicting increased or decreased risk of mortality. Several postoperative PBBMs were correlated with OS. Higher relative lymphocytes (HR 0.960), total albumin (HR 0.199, *P* < .0001), and PNI (HR 0.855, *P* = .0003) were predictive of decreased mortality. Higher relative neutrophils (HR 1.033, *P* < .0001) and PLR (HR 1.042, *P* < .0003) were predictive of increased mortality.

**Table 6:** Univariate Analysis of Preoperative and Postoperative Peripheral Blood Biomarkers (PBBMs) on Overall Survival in Total Cohort

	Preoperative			Postoperative		
PBBM	HR	95% CI	*P*‐value	HR	95% CI	*P*‐value
Relative neutrophil	0.986	0.970‐1.003	.096	**1.033**	1.016‐1.050	<.0001
Absolute neutrophil	1.000	0.999‐1.000	.497	**1.000**	1.000‐1.000	<.0001
Relative lymphocyte	1.011	0.994‐1.025	.202	**0.960**	0.940‐0.979	<.0001
Absolute lymphocyte	1.000	1.000‐1.000	.226	1.000	0.999‐1.000	.456
Relative monocyte	0.998	0.966‐1.002	.528	1.001	0.998‐1.002	.565
Absolute monocyte	1.000	0.999‐1.001	.363	**1.001**	1.001‐1.002	.0006
Relative eosinophil	1.057	0.943‐1.169	.325	0.995	0.978‐1.004	.351
Absolute eosinophil	1.000	0.999‐1.001	.949	0.999	0.997‐1.000	.192
Platelets	1.000	1.000‐1.000	.326	**1.000**	1.000‐1.000	.048
Total albumin	0.830	0.582‐1.142	.266	**0.199**	0.130‐0.308	<.0001
PNI	1.007	0.982‐1.023	.542	**0.855**	0.817‐0.895	<.0001
PLR	1.001	0.999‐1.002	.536	**1.042**	1.021‐1.060	.0003
NLR	0.997	0.946‐1.041	.915	**1.003**	1.002‐1.004	<.0001

Abbreviations: CI, confidence interval; HR, hazard ratio; NLR, neutrophil‐to‐lymphocyte ratio; PLR, platelet‐to‐lymphocyte ratio; PNI, Prognostic Nutritional Index.

### Multivariate Analysis of PBBMs Associated With Survival in cHNSCC

On multivariate analysis adjusted for AJCC T category (HR 1.309, *P* = .0003, 95% CI 1.136‐1.501), tobacco use (HR 1.709, *P* = 0.0006, 95% CI 1.253‐2.362), and history of chronic immunosuppression (HR 1.425, *P* = .070, 95% CI 0.970‐2.034), CCI (HR 1.137, *P* = .034, 95% CI 1.010‐1.278) was the only predictor of worse OS. All other tested variables included in the multivariate analysis (age, preoperative relative neutrophils, postoperative relative neutrophils and lymphocytes, postoperative platelets, postoperative absolute neutrophils and monocytes, postoperative total albumin, postoperative PNI, postoperative NLR, and postoperative PLR) were not significant predictors of OS on the multivariate level (Table 7).

**Table 7:** Multivariate Analysis of Significant Independent Factors in Overall Survival, Adjusted for Smoking, Immunosuppression, and AJCC T Category

Factor	HR	95% CI	*P*‐value
Age	1.028	0.994‐1.064	.109
CCI	**1.137**	1.010‐1.278	.034
Preoperative relative neutrophils	1.019	0.986‐1.051	.334
Postoperative relative neutrophils	1.021	0.962‐1.126	.613
Postoperative absolute neutrophils	1.000	1.000‐1.000	.060
Postoperative relative lymphocytes	1.065	0.984‐1.179	.163
Postoperative platelets	1.000	1.000‐1.000	.198
Postoperative absolute monocytes	1.001	0.999‐1.002	.549
Postoperative total albumin	2.161	0.074‐139.600	.834
Postoperative PNI	0.795	0.524‐1.14	.244
Postoperative NLR	0.926	0.834‐1.020	.049
Postoperative PLR	1.004	0.999‐1.010	.100

Abbreviations: CI, confidence interval; HR, hazard ratio; NLR, neutrophil‐to‐lymphocyte ratio; PLR, platelet‐to‐lymphocyte ratio; PNI, Prognostic Nutritional Index.

### Survival Analysis of Patients With and Without Laboratory Testing

Survival analysis of those with and without preoperative labs revealed no significant difference (*P* = .189) in OS; however, there was a significantly (*P* < .0001, HR 2.369, 95% CI 1.760‐3.189) higher OS in those who did not have postoperative PBBMs collected compared to those who did have postoperative labs available.

### Current Staging Systems

**Figure 1:**
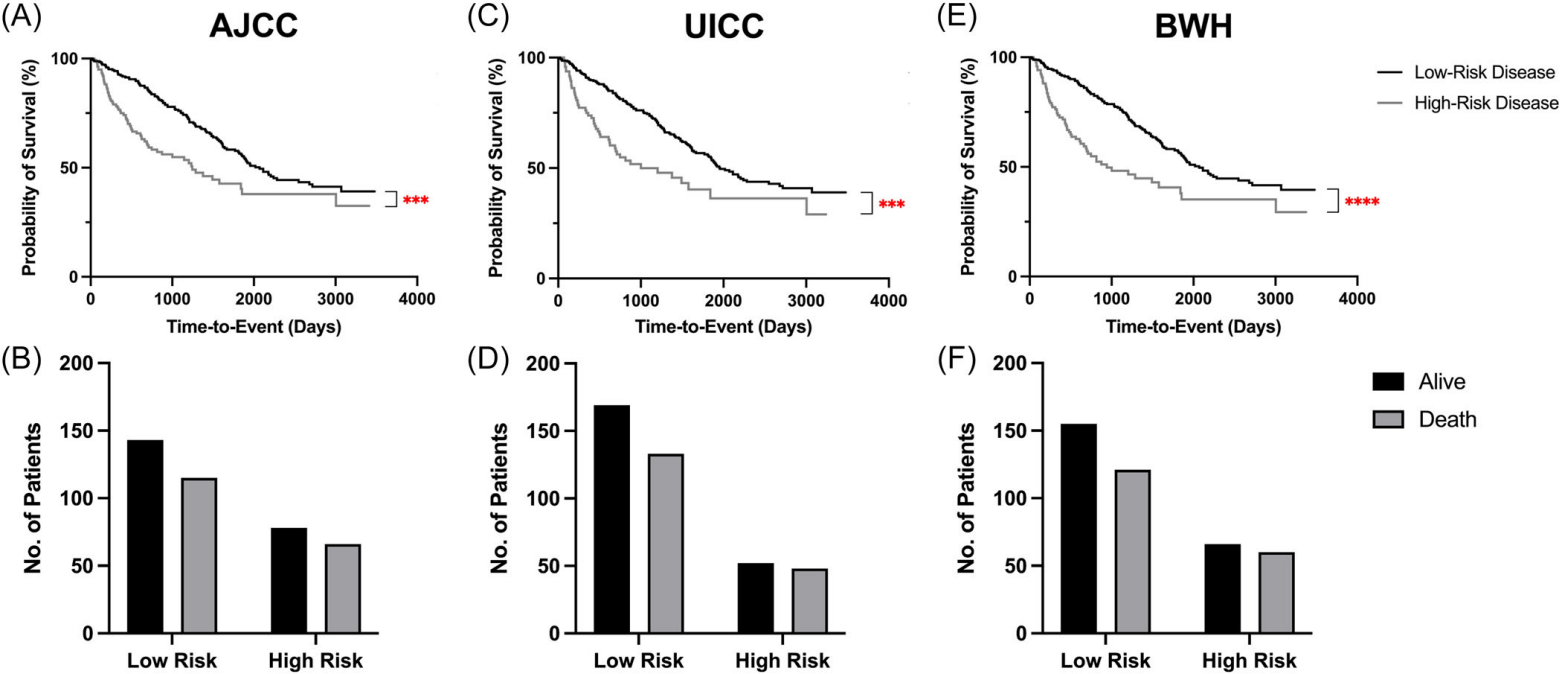
Survival analysis by low‐ and high‐risk staging. ***P < .001. ****P < .0001. Survival curves and count of AJCC (A, B), UICC (C, D), and BWH (E, F) by severity. Low‐risk was defined as AJCC/UICC T2 and BWH T2a and below.

On Log‐rank (Mantel‐Cox) tests of the constructed Kaplan‐Meier survival curves for all 3 T staging systems, AJCC (*P* = .0005, HR 1.760, 95% CI 1.255‐2.469), UICC (*P* = .0005, HR 1.874, 95% CI 1.261‐2.784), and BWH (*P* < .0001, HR 1.978, 95% CI 1.375‐2.874) all had significant differences in OS of their defined stages, both when grouped by individual stages and when grouped by low and high‐risk disease approximation (Table 1 and Figure 1). Only 36.5% (95% CI 29.8%‐43.7%), 26.5% (95% CI 20.6%‐33.4%), and 33.1% (95% CI 26.7%‐40.3%) of deaths were captured by high‐risk disease stratification in the AJCC, UICC, and BWH staging systems, respectively (Table 8). Homogeneity evaluation of whether those with same‐stage disease have similar OS outcomes revealed AJCC (*P* = .973), UICC (*P* = .791), and BWH (*P* = .652) had no significant difference in outcomes in their respective staging stratification. Monotonicity evaluation of the stepwise progression in worse outcomes with advancing disease staging revealed the AJCC (*P* = .748), UICC (0.621), and BWH (*P* = .684) systems did not show significant progression of worse OS with higher‐stage disease.

**Table 8:** Survival Outcomes in Low‐ and High‐Risk Disease per Staging System

System and stage	No. survivals (%)	No. death (%)	% Total system deaths (95% CI)
AJCC T1/T2	143 (55.4%)	115 (44.6%)	63.5% (56.3%‐70.2%)
AJCC T3/T4	78 (54.2%)	66 (45.8%)	36.5% (29.8%‐43.7%)
UICC T1/T2	169 (66.0%)	133 (44.0%)	73.5% (66.6%‐79.4%)
UICC T3/T4	52 (52.0%)	48 (48.0%)	26.5% (20.6%‐33.4%)
BWH T1/T2	155 (56.2%)	121 (43.8%)	66.9% (59.7%‐73.3%)
BWH T3/T4	66 (52.4%)	60 (47.6%)	33.1% (26.7%‐40.3%)

Abbreviation: CI, confidence interval.

## Discussion

PBBMs show promise as biomarkers for other cancers, but data are lacking specifically for cHNSCC. In the first known study attempting to identify relationship of preoperative and postoperative PBBMs to both intraoperative findings of cHNSCC and overall survival, we identified several markers that could better serve the clinician in conjunction with traditional staging systems in determining disease status, prognosis, and possible adjuvant therapy needs. Our study revealed no independently significant preoperative PBBMs that predicted OS in this cohort; however, postoperative PBBMs may correlate with OS. This conflicts with the current data on mucosal HNSCC, many of which demonstrate various preoperative PBBMs are significant predictors of OS. On the multivariate level, however, and when adjusted for both AJCC T category and known mortality impact variables like smoking and chronic immunosuppression, there were no significant PBBMs in predicting OS. There are likely several reasons for the lack of significant PBBMs in this cohort: the first may be due to the limitations with lack of blood collection within the 6‐month collection timeframe established in our methodology, as well as the reason for collection. Many lab values used were collected due to some other indication or hospitalization. Another possibility is the lack of measuring disease‐specific outcome, as OS in this cohort is confounded by other comorbid diseases with to advanced age. While it could be true that PBBMs are unreliable to prognosticate cHNSCC outcomes, it is unlikely to be the case and cannot be determined from a retrospective review alone. Further investigation in a prospective nature with standardized blood collections would be optimal in determining true validity of PBBM use in cHNSCC care.

While staging drives treatment recommendations for many cancer patients, given the advanced age for many cHNSCC patients, age plays an important factor in decision‐making as well. As expected, increased age was a significant independent risk factor for mortality on the univariate scale (Table 3), although it was not significant for predicting OS on multivariate analysis in this cohort (Table 7). CCI significantly predicted worsened OS on both the univariate and multivariate level (Tables 3 and 7). This is consistent with previous reports in the literature demonstrating worsened outcomes with greater levels of comorbidity.^20^ CCI and other calculations, including frailty scores, could provide better estimations of outcomes when determining candidacy for treatments in cHNSCC instead of age alone.

There has been interest in the development of more specific personalized or “precision” oncology approaches to complement treatment decision making, such as the use of tumor sequencing and gene expression profiling.^21^ If broadly applied however, there are significant cost and access considerations to these diagnostic techniques currently. PBBMs have thus been studied in other cancer types as a form of more easily accessible and cost‐effective monitoring that could be used in the diagnostic, treatment, and surveillance stages. Our study shows mixed results regarding PBBMs and cHNSCC however. In our cohort, tumor size correlated positively with relative and absolute neutrophils, absolute lymphocytes, absolute monocytes, platelets, and PLR; it correlated negatively with albumin (Table 4A). Total metastatic lymph nodes and metastatic‐to‐total lymph nodes positively correlated to relative monocytes and PLR, respectively. Deep invasion and perineural invasion were independently associated with both higher absolute neutrophils and platelets (Table 4B,C). These findings are not inherently surprising; firstly, the inflammatory response generated by the tumor microenvironment is generalized by increased nonspecific markers, like platelets, neutrophils, and monocytes, so a positive correlation with more advanced disease was expected.^22^, ^23^ Negative correlation with albumin was also expected given this has been shown in prior literature to decrease with extended periods of inflammation.^24^ These relationships have been demonstrated in various solid tumors, including mucosal head and neck squamous cell carcinoma, non‐small cell lung cancer and melanoma.^8^, ^9^, ^11^, ^25^, ^26^ Further validation of these findings could suggest that preoperative laboratory testing may be beneficial for guiding planning of treatment extent, such as considering sentinel lymph node biopsy or elective neck dissection when preoperative tumor assessment alone may not have suggested nodal basin management.

As the main goal of this study was to determine if PBBMs could predict survival in our cohort, we also aimed to evaluate the three main staging systems' ability to predict our cohort's survival. All three staging systems demonstrated significantly different survival curves amongst their stages (Figure 1A,C,E), although BWH best predicted OS. Surprisingly, each staging system applied to our cohort was not effective in separating out patients by survival (Table 8). Further analysis revealed that while there was significant separation of survival over time in each of the three systems, there was no significant ability to determine monotonicity and homogeneity. The capacity for a staging system to demonstrate both a stepwise progression of worse outcomes with increasing stage (monotonicity) and similar outcomes for those in the same stage (homogeneity) is crucial for the prognostic capability of any staging system. This is likely confounded by the fact our cohort is of advanced age with high comorbidity burden and may be more likely to have a poor prognosis from another disease outside of cHNSCC and further limited by a single‐institution review of disease outcomes.

### Limitations

As a retrospective review, there are inherent limitations in availability of data consistency and quality. The first and most notable was lack of laboratory recordings at consistent intervals and lack of routine lab follow‐up. Only 67% and 63% of our cohort had preoperative and postoperative labs, respectively, reported within the accepted time frame. Furthermore, our survival analysis between patients with and without labs did show a significant difference between the postoperative groups. We theorize this may be due to the previously discussed confounding events of other infections, diseases, or postoperative complications that elicited further testing. Many of these lab draws may have also been unnecessary; in fact, our analysis showing a higher OS for patients who did not have postoperative PBBMs collected suggests that there may be unmeasured confounders for patients receiving postoperative PBBMs that influence survival. Future studies to validate these findings should attempt a more prospective approach to ensure laboratory values are less reflective of underlying comorbidities or other acute health conditions. While our cohort is one of the largest well‐described cHNSCC cohorts, the study may be still be underpowered to detect certain effects. Cause of death in most cases was unknown, and whether it was related to, secondary from, or at all caused by the associated disease was difficult to assess. Granularity of follow‐up data was not robust enough for analysis of progression‐free and disease‐free survival in this cohort. Future studies should verify these results with disease‐specific outcomes.

## Conclusions

Perioperative PBBMs are potential biomarkers for cHNSCC disease characteristics such as size, metastasis, and degree of invasion, as well as OS. Future work should be done in a prospective setting with stratification of high‐risk disease to verify these data and whether this can aid decision‐making for treatment planning and prognostic discussions.

## Author Contributions

**Mikayla G. Hubbard**, conceptualization of project, data collection and analysis, statistical analysis, manuscript drafting and revision, final approval of manuscript, oral presentation; **Emily H. Chestnut**, data collection and analysis, manuscript drafting and revision, final approval of manuscript; **Meghana C. Bhaskara**, conceptualization of project, manuscript drafting and revision, final approval of manuscript; **Lena W. Chen**, data collection and analysis, statistical analysis, manuscript drafting and revision, final approval of manuscript; **Elena Kennedy**, statistical analysis, manuscript drafting and revision, final approval of manuscript; **Aimee Lee**, data collection and analysis, final approval of manuscript; **Jessica Yesensky**, conceptualization of project, manuscript drafting and revision, final approval of manuscript, project supervision, drafting of presentation; **Janice L. Farlow**, conceptualization of project, manuscript drafting and revision, project supervision, final approval of manuscript, drafting of presentation.

## Disclosures

### Competing interests

None.

### Funding source

None.
